# The Role of the Gut Microbiome Dysbiosis in Metabolic Dysfunction: A Mini Review

**DOI:** 10.3390/healthcare13233029

**Published:** 2025-11-24

**Authors:** Amani N. Shafik, Veronia F. Fahim, Fady A. Iskander, Hassan A. Elsayegh, Hani Serag, Hanaa S. Sallam

**Affiliations:** 1 Medical Pharmacology Department, Faculty of Medicine, Cairo University, Giza 11956, Egypt; amaninabil@kasralainy.edu.eg (A.N.S.); veronia.fawzy@kasralainy.edu.eg (V.F.F.); 2 Medical Pharmacology Department, Faculty of Medicine, Badya University, Giza 12566, Egypt; 3 Medical Physiology Department, Faculty of Medicine, Badya University, Giza 12566, Egypt; fady.adel@badyauni.edu.eg; 4 Medical Anatomy Department, Faculty of Medicine, Badya University, Giza 12566, Egypt; hassan.ahmed@badyauni.edu.eg; 5 Department of Population Health and Health Disparities, Division of Global Partnerships, University of Texas Medical Branch, Galveston, TX 77555, USA; hssallam@utmb.edu; 6 Medical Physiology Department, Faculty of Medicine, Suez Canal University, Ismailia 41522, Egypt

**Keywords:** brain health, gut–brain axis, metformin, metabolic dysfunction, microbiota

## Abstract

**Highlights:**

**What are the main findings?**

- In addition to its role in regulating glucose metabolism, it also has an emerging potential to modulate the gut microbiome beneficially; accordingly, Metformin may indirectly support improved brain health.

**What are the implications of the main findings?**

- Metformin has a promising therapeutic role in promoting cognitive and neurological well-being.

**Abstract:**

This review provides a comprehensive overview of the complex and dynamic bacterial composition of the human gastrointestinal (GI) microbiota and explores its integral role in the microbiome–gut–brain axis. It discusses the physiological and molecular pathways through which the gut microbiota communicates with the central nervous system, highlighting key barriers that can impede effective signaling along this axis. The review also delves into the influence of microbiota on brain health, including cognitive function, mood regulation, and neuroinflammation. It considers how disruptions in this system—known as dysbiosis—can contribute to metabolic and neurological dysfunction. The central focus of the article is the role of the commonly prescribed antidiabetic drug metformin, not only in regulating glucose metabolism but also in its emerging potential to beneficially modulate the gut microbiome. By doing so, metformin may indirectly support improved brain health. Ultimately, the article seeks to inform both healthcare practitioners and patients about the promising therapeutic implications of microbiome-targeted strategies, particularly metformin, in promoting cognitive and neurological well-being.

## 1. Introduction

The gut microbiome is increasingly recognized as a key regulator of metabolic, inflammatory, and neurocognitive processes. The gut microbiome is increasingly recognized as a key regulator of metabolic and cognitive health [1,2,3,4]. A balanced microbial ecosystem maintains nutrient metabolism, glucose regulation, and intestinal integrity, whereas disruptions in diversity and function can predispose individuals to metabolic disorders. Evidence indicates that shifts in microbial composition emerge early in disease pathways, underscoring the microbiome’s role in shaping metabolic and cognitive health trajectories [1,2,3,5,6,7,8] and supporting the development of microbiome-targeted therapeutic strategies [9,10].

Gut dysbiosis, characterized by reduced beneficial taxa and increased pathogenic groups, has been consistently linked to impaired glucose homeostasis. Dysbiosis promotes intestinal permeability, endotoxemia, and chronic low-grade inflammation, all of which contribute to insulin resistance [1,3,4].

In addition, altered microbial composition can influence neurodegenerative and cognitive disorders through effects on gut–brain axis signaling. Changes in microbial metabolites, including short-chain fatty acids and bile acids, can modulate neuroinflammation, microglial activation, and metabolic signaling along the gut–brain axis, contributing to cognitive decline and neurological disorders. These disruptions were linked to the pathogenesis of Alzheimer’s disease, Parkinson’s disease, and mild cognitive impairment, with microbial profiles increasingly recognized as indicators of disease severity [5,7,11,12].

Metformin, the first-line therapy for type 2 diabetes, exerts microbiome-mediated actions in addition to reducing hepatic glycogenolysis and gluconeogenesis, improving insulin sensitivity, and overall glycemic control. Metformin modulates gut microbial composition and activity, influencing short-chain fatty acid synthesis and bile acid metabolism, increasing beneficial taxa, and shifting metabolic pathways [13,14,15,16]. These microbiome-mediated actions have stimulated interest in its potential neuroprotective properties and its role as a therapeutic link between metabolic and brain health.

This review summarizes current evidence on microbiome–brain interactions and highlights how metformin-induced microbial changes may influence neuroinflammatory and metabolic pathways underlying cognitive function (Table 1). Understanding these mechanisms may guide future strategies to target the gut microbiome for metabolic and neurological resilience.

## 2. Methods

This narrative review aimed to explore current perspectives and conceptual developments related to the role of gut microbiome dysbiosis in metabolic dysfunction. Rather than using a systematic search strategy, we adopted a purposive, selective approach to identify literature relevant to the review’s thematic focus.

Sources were identified primarily through searches of PubMed, Google Scholar, and relevant journals using key terms such that combines Gut Microbiome Dysbiosis and Metabolic Dysfunction.” Articles were selected based on their conceptual relevance, publication in peer-reviewed journals, and contribution to the topic’s development. Priority was given to influential publications, recent developments, and works frequently cited in the field. Reference lists of selected articles were also reviewed to identify additional sources. The literature included original research articles, policy documents, expert opinions, and narrative commentaries. The synthesis of findings was organized thematically to highlight recurring patterns, evolving concepts, and areas of debate or uncertainty.

Given the non-systematic nature of the review, findings are intended to provide a conceptual overview rather than a comprehensive assessment of all available evidence. Potential selection bias and subjectivity are acknowledged as inherent limitations of the narrative approach.

## 3. Gut Microbiota

This term refers to the various microorganisms residing in the human intestine, whose collective genomes and metabolites form the microbiome [3,30]. Dominant phyla include *Bacteroidetes* and *Firmicutes*, which produce short-chain fatty acids (SCFAs) critical for gut integrity and host metabolism [31,32]. These microbes assist in digesting nutrients, synthesizing vitamins, and regulating immune responses and metabolic pathways. They also protect against pathogens by maintaining epithelial barriers and producing antimicrobial compounds [33]. Disruptions in microbial composition (i.e., dysbiosis) contributed to various diseases, prompting interest in probiotics for therapeutic use [9].

## 4. Gut–Brain Axis (GBA)

The gut microbiota plays a critical role in the GBA. Microbe-derived signals reach the central nervous system (CNS) either directly via systemic circulation or indirectly via receptors on enteroendocrine cells (EECs), and enterochromaffin cells (ECCs) in the gut, or the mucosal immune system [34]. The signals, in turn, trigger a CNS response through the autonomic nervous system (ANS) and the hypothalamic–pituitary–adrenal (HPA) axis [35]. Strong evidence of microbiota and brain linkages in clinical practice is that gut dysbiosis has been causally associated with neurological illnesses such as autism and anxiety-depressive behaviors, as well as functional gastrointestinal disorders, including irritable bowel syndrome [36,37]. Numerous communication channels, including immunological, endocrine, neuronal, and metabolic pathways, have been identified along the GBA [38,39,40,41].

### 4.1. Microbiota and Neurotransmitter Synthesis

Various neurotransmitters, such as acetylcholine, noradrenaline, serotonin, and dopamine, are synthesized by numerous bacterial species residing in the gastrointestinal tract [22,23,42,43], contribute to the intricate communication network between the gut and the brain [44]. Gamma-aminobutyric acid (GABA) is synthesized by *Bifidobacterium* and *Lactobacillus* species; Noradrenaline is synthesized by the species *Bacillus*, *Escherichia*, and *Saccharomyces*; serotonin by *Streptococcus*, *Escherichia*, *Candida*, and *Enterococcus* species, while acetylcholine is synthesized by *Lactobacillus*, and dopamine by *Bacillus* [23]. These microbiota-generated neurotransmitters cross the intestinal mucosal barrier; however, their effects on brain function are thought to be mediated indirectly via interactions with the enteric nervous system (ENS) [4,45].

### 4.2. Microbiota and Enteroendocrine Signaling

Bacterial metabolites interact with the gut epithelium, prompting EECs to create active amines through the intracellular decarboxylation of active amine precursors. These are subsequently deposited in secretory vesicles [46]. EECs are considered crucial sensors of gut microbiota and/or microbial metabolites, playing a vital role in maintaining mucosal immunity and gut barrier function, as well as in visceral hyperalgesia and gastrointestinal (GI) motility in health and disease [41,47,48,49].

The brain controls the functions of intestinal effector cells, including enteric neurons, interstitial cells of Cajal, immune cells, epithelial cells, smooth muscle cells, and enterochromaffin cells, through a combination of neural and hormonal signaling. On the other hand, the gut microbiota, which is involved in the gut–brain reciprocal connections, also influences these same cells. It is currently emerging that a microbiome GBA exists [35].

### 4.3. Microbiota Metabolites

The gut microbiota produces a remarkably diverse array of metabolites derived from the anaerobic fermentation of dietary components and endogenous chemicals produced by both the host and microbes [50]. Many of these resulting compounds hinder the growth of their rivals, thus maintaining the diversity of commensal species and eliminating pathogenic bacteria [51]. Furthermore, these gut microbiota metabolites play roles in a variety of key physiological processes, including host energy metabolism and immunology, as well as other unknown activities, which together compose the human metabolome [52].

Colonic bacteria enzymatically break down complex carbohydrates, producing SCFAs (e.g., for most common SCFAs are propionate, butyrate, and acetate with a 1:1:3 ratio [17,18]. SCFAs, quickly absorbed by epithelial cells, regulate cellular processes (e.g., gene expression, chemotaxis, differentiation, proliferation, and cell death) [19].

## 5. Barriers to Microbiota-Gut–Brain (MGB) Signaling

The intestinal barrier and the BBB are the two primary obstacles to MGB signaling. Gut flora, inflammatory signals, and stress influence the permeability of both dynamic barriers. In a healthy state, these tight barriers inhibit the transmission of microbiome-related immunological signals to the brain [53,54].

### 5.1. Intestinal Barrier

The intestinal barrier has multiple layers: (1) an outer layer of mucus, commensal gut flora, and defense molecules, including secretory immunoglobulin A (sIgA) and antimicrobial proteins (AMPs), (2) an intermediate layer of intestinal epithelial cells (IECs), and (3) an inner sterile layer of innate and adaptive immune cells [55]. The inner layer protects IECs through physical separation and innate immune mechanisms [56].

The mucus layer includes (1) a loose outer layer (lumen-facing) and (2) an interior dense layer anchored to the epithelium. Commensal microorganisms inhabit the outer layer, where they form a biofilm and utilize glycans in the absence of dietary fiber [57]. Gut-microorganisms-induced SCFAs play an essential role in preserving intestinal structural integrity by maintaining tight cell junctions and limiting the activation of gut-associated immune cells [58].

Low-fiber Western diets or chronic stress thin/shrink the mucus layer, thereby increasing intestinal permeability to microorganisms [59,60]. Commensal bacteria cell wall components activate TLRs on dendritic cell extensions, inducing cytokine release and stimulating gut-associated immune system. These cytokines may weaken IEC’s tight connections, allowing microorganisms to cross the intestinal barrier, via microfold cells, and enter systemic circulation, leading to metabolic endotoxemia [61].

### 5.2. Blood–Brain Barrier (BBB)

The BBB controls the transport of substances from the circulatory system to the cerebrospinal fluid [62]. The BBB is composed of capillary endothelial cells, astrocytes, and pericytes. Tight junction proteins, which limit the paracellular transport of water-soluble compounds from the blood to the brain [63], are primarily composed of transmembrane proteins such as claudins, tricellulin, and occludin [64].

**Figure 1:**
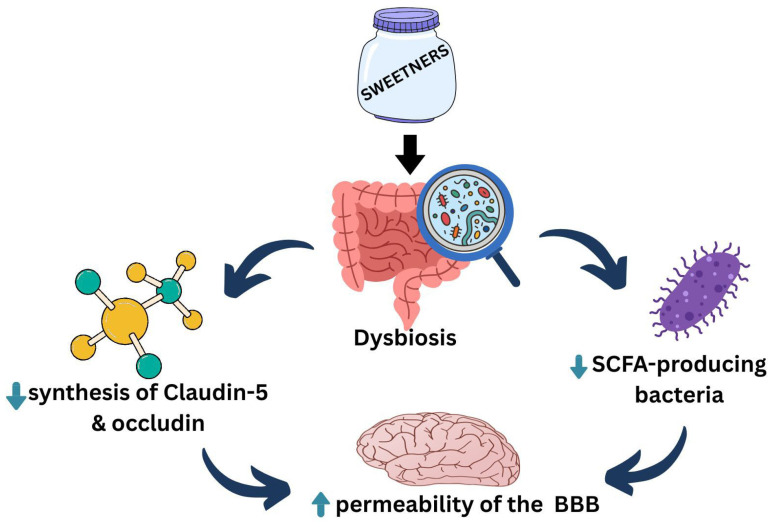
This diagram shows the effect of sweeteners on the gut microbiome and the Gut–Brain axis. Upward teal arrow signifies increase; downward teal arrow signifies decrease. BBB: Blood–Brain Barrier—SCFA: Short-Chain Fatty Acid.

The gut microbiota, which modulates the intestinal barrier, may affect BBB permeability. The gut microbiome enhances the synthesis of tight junction proteins such as Claudin-5 and occludin, thereby reducing BBB permeability [65]. The SCFA-producing bacteria colonizing the gut also reduces the permeability of the BBB, indicating that SCFAs play a crucial role in the development and maintenance of the BBB [66]—Figure 1.

## 6. Role of the Gut Microbiota in Health

With its extensive genetic makeup and metabolic diversity, the gut microbiota offers several beneficial characteristics to the host. Among these bacteria’s most significant functions are their assistance in preserving the mucosal barrier’s integrity, their provision of nutrients, including vitamins, and their defense against infections. Furthermore, for the immune system to operate properly, commensal microbiota and the mucosal immune system must interact [67].

Alterations in gut bacterial composition and disruption of gut homeostasis, which have been linked to the etiology of gut–brain diseases, are frequently brought on by dietary patterns, antibiotic use, and viral and bacterial infections [68].

SCFAs are signaling molecules produced only by gut microorganisms during the fermentation of dietary fiber, since humans lack the enzymes necessary to digest fiber [69]. Once absorbed by colonic epithelial cells, SCFAs activate free fatty acid 2 (FFA2), FFA3, GPR109a, and Olfr78 receptors and act on a variety of targets. SCFAs have been linked to various physiological processes, including neuroplasticity, gene expression, dietary intake, and immune system modulation [24,70].

GI microbiota is essential to the development of the intestinal mucosal and the systemic immune systems [71,72]. GI microbiota are also vital for the de novo production of vitamins, including vitamin K, riboflavin, biotin, nicotinic acid, pantothenic acid, pyridoxine, and thiamine [20,73]. Lactic acid bacteria create vitamin B12 [74] and bifidobacteria, as the primary providers of folate, contribute to DNA synthesis and repair [21].

## 7. Role of GBA in Health

Bacterial colonization of the gut is essential for the formation and maturity of both the ENS and the CNS. The lack of microbial colonization leads to changes in gene expression and neurotransmitter turnover in both the CNS and ENS. Additionally, it alters gut sensory-motor functions, leading to delayed gastric emptying and intestinal transit [35].

Microbiota regulates the HPA axis set point, which generates glucocorticoids such as cortisol in humans and corticosterone in rodents, which modulates stress reactivity and anxiety-like behavior [75]. Several mental disorders and behavioral changes have been linked to alterations in the HPA axis [76,77].

Cortisol influences the MGB axis Via several pathways. Its receptors are on epithelial cells, immune cells, and EECs, suggesting a direct impact of cortisol on gut function [35,78,79]. Cortisol can also alter gut transit time, intestinal permeability, and nutrient availability, which, in turn, can influence gut microbiota, subsequently shaping its diversity and composition. Central effects of cortisol occur by binding to glucocorticoid receptors in the prefrontal cortex, amygdala, and hippocampus. Additionally, there is evidence that microorganisms in the stomach can trigger stress circuits in the CNS and ENS via the vagus nerve and sensory neurons, respectively.

Microbiota also regulate serotonergic pathways in the limbic system [35,80,81]. It has a strong influence on the EECs, which synthesize and secrete around 90% of serotonin. Furthermore, the gut microbiome has been discovered to influence hippocampal serotonin levels, possibly by affecting the peripheral availability of tryptophan [82]. Gut-derived serotonin cannot penetrate the BBB, but serotonin derivatives (N-acetyl serotonin and melatonin) and their precursor (5-hydroxytryptophan) can, and can affect the CNS [83].

Stress, anxiety, and violence are all triggered by serotonin (5-HT). Research has shown that disorders of gut microbes and the serotonergic system significantly influence the etiology of neuropsychiatric and neurological illnesses [84]. They are linked to numerous CNS diseases, such as Alzheimer’s disease [85], Parkinson’s disease [86], and amyotrophic lateral sclerosis [87]. As a result, gut microbe disorders are thought to be a major cause of dementia.

## 8. Dysbiosis of the Gut Microbiome Contributes to Metabolic Dysfunction

The gut microbiota is a major environmental factor that regulates human metabolism and contributes to the development of chronic illnesses such as obesity, diabetes, and atherosclerosis [88]. Reported causes include increases in systemic LPS, changes in bile acid metabolism [89], changes in SCFAs synthesis [19,89,90], changes in gut hormone secretion [91,92,93] and changes in circulating branched-chain amino acids [94,95,96].

### 8.1. Lipopolysaccharide (LPS)

LPS, or endotoxin, is a bacterial cell wall component primarily found in Gram-negative bacteria that triggers an inflammatory response by activating TLR-4 and transforming growth factor (TGF)-mediated pathways [97,98]. Increases in systemic LPS or lipoprotein binding protein have been linked to low-grade, chronic inflammation in obesity [99], metabolic syndrome [100], and type 2 diabetes [101].

Several potential routes by which the gut microbiota may influence circulating LPS levels. These include: (1) Changes in the balance and types of intestinal bacteria can affect LPS bioavailability. For example, in diabetes, dysbiosis is characterized by a reduction in butyrate-producing, LPS-lacking, Gram-positive Clostridial species, and an increase in LPS-containing, Gram-negative opportunistic pathogens, including certain *Bacteroidetes* and *Proteobacteria* species [6,102,103]. (2) Increased intestinal permeability, often referred to as “leaky gut,” can enable LPS to move through intercellular pathways. The gut microbiome plays a key role in regulating gut permeability by supporting the health of intestinal cells, maintaining their tight junctions, and preserving a protective mucous layer. This regulation is partly achieved by supplying nutrients, such as SCFAs, to the epithelial cells [104,105,106].

Probiotics, such as *Streptococcus thermophilus* and *Lactobacillus acidophilus*, have been shown to prevent TNF-α and Interferon Gamma (IFγ)-induced increase in human intestinal epithelial cells’ permeability in vitro. This highlights the vital role certain bacteria play in maintaining a healthy intestinal barrier [28,29].

### 8.2. Bile Acids

The gut microbiome plays a key role in bile acid metabolism. In the liver, bile acids are synthesized from cholesterol. Glycine or taurine-conjugated bile acids form bile salts, which are secreted into the small intestine. While 95% of bile salts become reabsorbed and transported back to the liver via the enterohepatic circulation, 400–600 mg reach the colon, to be converted by anaerobic bacteria to secondary bile acids, which exert widespread effects, including on the brain [26,107].

Secondary bile acids activate the ileal farnesoid X receptor (FXR) [108,109], which stimulates the production of fibroblast growth factor 19 (FGF19). FGF19 then enters the bloodstream, crosses the BBB, and activates the arcuate nucleus of the hypothalamus [110]. This hypothalamic activation enhances glucose metabolism regulation and reduces HPA axis activity. Vertical sleeve gastrectomy, a type of bariatric surgery, has been shown to rely on FXR signaling for its anti-diabetic effects [111]. Similarly, an intestinal FXR agonist has been demonstrated to improve insulin sensitivity [112]. In the pancreas, FXR activation influences insulin transport and secretion [113] and may also protect islets against lipotoxicity [114].

Ileal L cells express Takeda G protein-coupled receptor 5 (TGR5), which is activated by secondary bile acids. These secondary bile acids are exclusively produced by intestinal bacteria, and their levels are influenced by the composition of the gut microbiota [115]. Activation of TGR5 enhances glucose homeostasis by stimulating L cells to release glucagon-like peptide-1 (GLP-1). This increase in GLP-1 regulates ingestive behavior and food intake [27].

### 8.3. Short Chain Fatty Acid (SCFAs)

Nondigestible carbohydrates are fermented by bacteria in the colon to produce SCFAs, with butyrate, acetate, and propionate being the main products. Dietary fiber content, microbiota, and SCFAs interact because diets high in oligosaccharides change the makeup of microorganisms, produce more SCFAs, and lower the pH of the luminal fluid [24,70].

Preclinical and clinical research have demonstrated that the synthesis of SCFAs induces the ileum’s L cells to release the satiety hormone GLP-1, resulting in behavioral changes and altered satiety perceptions [116]. Additionally, SCFAs influence the synthesis of 5-HT (serotonin) in ECCs [117]. By stimulating AMP kinase and free fatty acid receptors 2 and 3 (FFAR2 and 3), sometimes referred to as G-protein coupled receptors 43 and 41 [118], SCFAs behave as signaling molecules. SCFAs were shown to prevent de novo development of non-alcoholic fatty liver disease by stimulating fatty acid oxidation [25].

More attention has been paid to butyrate as a possible helpful intermediary; in people with diabetes, butyrate-producing bacteria are less prevalent [119,120]. Supplementing mice with butyrate increased their insulin sensitivity.

### 8.4. Gut Hormone Secretion

The release of gut hormones like GLP-1 and peptide YY (PYY), which regulate energy balance and glucose metabolism, is linked to SCFAs [91]. In response to dietary intake, proglucagon undergoes tissue-specific processing to produce GLP-1, which enhances insulin secretion from pancreatic β-cells. Both GLP-1 and PYY act in the hypothalamus to suppress food intake [121]. These hormones are also thought to contribute to the metabolic benefits observed after gastric bypass surgery, with GLP-1 playing a role in metformin’s glucose-lowering effect [122,123].

PYY, like GLP-1, is synthesized by L-cells in the ileum and colon and regulates satiety by activating Agouti-related peptide (AgRP) neurons in the hypothalamus and Y2 receptors on neuropeptide Y (NPY). This suppresses appetite by disinhibiting the satiety-inducing proopiomelanocortin/alpha-melanocyte-stimulating hormone (POMC/α-MSH) pathway [124]. The gut microbiota’s influence on PYY secretion is significant for understanding obesity and metabolic diseases [125]. Additionally, secondary bile acids stimulate PYY secretion via pathways like those for GLP-1 [126].

### 8.5. Microbial Synthesis of Amino Acids

Human microbes participate in amino acid synthesis and affect serum amino acid levels [127]. Bacteria may serve as a source of branched-chain amino acids (BCAAs), as they are more abundant in bacterial cells than in eukaryotic cells. Notably, bacteria can synthesize all 20 amino acids required for protein production. Various lines of evidence suggest that gut microbiota influence the levels of amino acids absorbed by the host and the composition of the host’s amino acid pool. Circulating amino acids help maintain glucose homeostasis by promoting the release of insulin and glucagon. BCAAs, in particular, appear to have a distinct role in glucose regulation, which may be linked to an increased risk of diabetes. In fact, plasma concentrations of five branched-chain and aromatic amino acids (isoleucine, leucine, valine, tyrosine, and phenylalanine) were shown to predict the development of diabetes, independent of traditional risk factors [128].

## 9. The Role of Metformin in Modulating the Gut Microbiome

**Figure 2:**
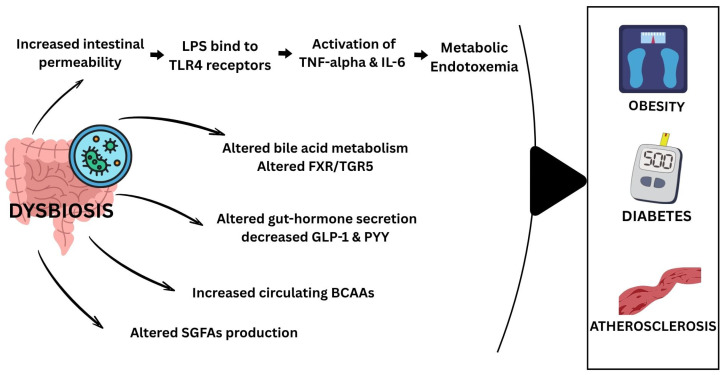
This diagram shows the effect of dysbiosis of the Gut Microbiome and its contribution to Metabolic Dysfunction. BBB: Blood–Brain Barrier—SCFA: Short-Chain Fatty Acid.

Table 2 summarizes the effects of metformin on microbiome regulation. Metformin was reported to alter the gut microbiota, which plays a crucial role in glucose metabolism and overall metabolic health [129]. Dysbiosis, marked by an imbalance between the harmful bacteria (e.g., *Proteobacteria* and *Firmicutes*) and the beneficial species (e.g., *Akkermansia muciniphila* and *Bifidobacterium* spp.), has been associated with impaired glucose metabolism and insulin resistance [130]—Figure 2.

### 9.1. Metformin Mechanism of Action

Metformin primarily acts by activating AMP-activated kinase (AMPK), which influences various cellular processes [136]. It lowers hepatic glucose production and improves insulin sensitivity [137]. Additionally, metformin exhibits strong anti-inflammatory and neuroprotective properties, potentially mediated by its effects on gut microbiota [138,139].

One of metformin’s key actions is maintaining intestinal barrier integrity, thereby reducing serum LPS levels and enhancing glucose metabolism by preventing the migration of pro-inflammatory factors. The drug also promotes SCFAs production, improving insulin sensitivity by modulating substrate metabolism in peripheral tissues. This mechanism entails an increase in the population of SCFAs-producing bacteria, reinforcing Metformin’s role in regulating glucose levels. Additionally, metformin regulates bile acid levels, thereby improving glucose metabolism. Studies have shown that it elevates plasma bile acid while altering gut microbiota to enhance metabolic outcomes. Its effect on gut microbiota composition, particularly by promoting beneficial bacteria such as *Akkermansia muciniphila*, plays a significant role in glucose homeostasis and overall metabolic health [32,140]. Metformin may influence glucose transfer from the intestinal lumen to the bloodstream and enhance glucose sensing in the gut. This highlights an additional mechanism by which metformin contributes to its glucose-lowering effects.

**Figure 3:**
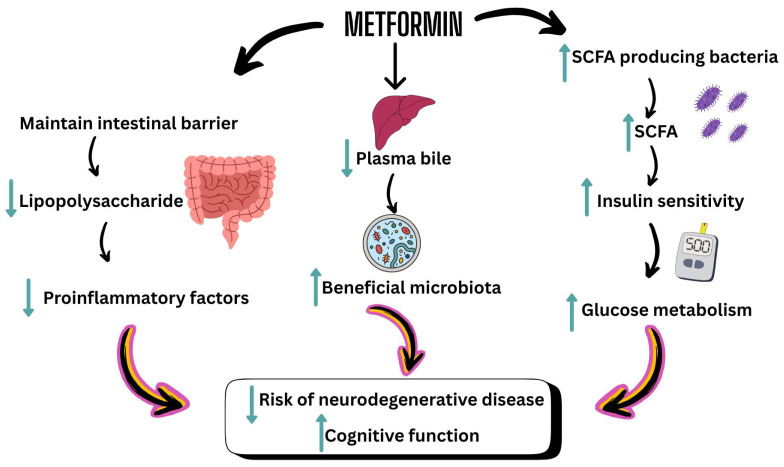
This diagram illustrates how metformin modulates the gut microbiome to improve human brain health. Upward teal arrow signifies increase; downward teal arrow signifies decrease. SCFA: Short-Chain Fatty Acid.

Metformin’s modulation of gut microbiota may have implications for brain health, potentially addressing cognitive disorders [138]. Through the GBA, metformin’s influence on gut microbiota opens the door to microbiome-targeted therapies that could offer cognitive benefits. These findings underline the importance of exploring metformin’s broader effects beyond its traditional role in metabolic regulation. Figure 3 shows the role of Metformin in modulating the gut microbiome to improve human brain health.

### 9.2. Effects of Metformin on Cognition

Metformin has been associated with considerably reduced risk of dementia and neurodegenerative disorders, as well as enhancements in three cognitive domains: memory, semantic memory, and executive function [141,142]. Such neuroprotective benefits may occur via improving neuronal AMPK-induced energy homeostasis.

Metformin treatment also effectively counteracted amyloid-beta-induced effects on human neural stem cells (hNSCs) by suppressing caspase activity and reducing cytosolic cytochrome c levels. Furthermore, co-treatment with metformin played a key role in restoring mitochondrial structure in affected stem cells, bringing it closer to normal morphology [30]. AMPK activation induced by metformin protected the stem cells against cytotoxicity caused by advanced glycation end products [143].

Metformin reduced Alzheimer’s disease-associated alterations in differentiated mouse neuroblastoma cell lines, such as Neuro-2a [144]. It also inhibited tau phosphorylation in cultured neurons and in mouse brains. Metformin was additionally shown to prevent apoptotic cell death in primary cortical neurons [145] and restore the type 2 diabetes-induced decrease in cell proliferation and neuroblast differentiation in the dentate gyrus of the rat hippocampus [146].

Metformin also reduced the incidence rate of dementia in humans compared to those treated with sulfonylureas and thiazolidinediones. Combination therapy with metformin and thiazolidinedione lowered the risk of all-cause dementia. Combination therapy with metformin and sulfonylureas protected against all types of dementia over 2 years [147]. It has been suggested that accurate assessment of mitochondrial pathways as biomarkers would help in disease-stage-specific-metformin-targeted therapy, since mitochondrial dysfunction and related pathway disruptions contribute to the pathogenesis of neurological degenerative diseases [10].

## 10. Conclusions

The importance of microbiota in health cannot be overstated. Emerging research has highlighted the crucial role that the diverse community of microorganisms residing in our bodies plays in various physiological processes. These microbial populations play a crucial role in digestion, immune function, and the synthesis of essential nutrients.

Furthermore, microbiota influences the GBA, affecting mental health and cognitive function. Disruptions in microbial balance have been linked to a range of health issues, including gastrointestinal disorders, metabolic conditions, and even neurodegenerative diseases.

Metformin impacts gut microbiota composition, improving glucose regulation and potentially enhancing brain health. Since mitochondrial dysfunction contributes to various neurodegenerative diseases, precise measurements of altered mitochondrial pathways may help identify biomarkers to guide stage-specific metformin therapy. However, more research is needed to fully understand how metformin influences gut microbiota and its role in managing type 2 diabetes and cognitive function. Future studies should investigate the microbial changes associated with therapeutic benefits, paving the way for new treatments for metabolic and cognitive disorders.

## 11. Gap of Knowledge

Despite notable progress in understanding the interactions among gut microbiota, metabolic regulation, and the gut–brain axis, multiple unresolved questions remain in this field. The causal relationship between specific microbial taxa and host metabolic outcomes remains unconfirmed, mainly because most studies rely on correlation rather than mechanistic proof. Moreover, the precise molecular mechanisms by which metformin modulates gut microbial diversity, composition, and function, particularly its effects on SCFA synthesis, bile acid metabolism, and intestinal barrier integrity, remain incompletely elucidated. Interindividual differences in gut microbial responses to metformin remain insufficiently understood and may be influenced by genetic, dietary, and environmental factors. Additionally, only a few human studies have explored how Metformin-induced alterations in the gut microbiota contribute to improvements in cognitive and neurological function.

## 12. Limitations

While metformin and microbiome-targeted strategies show promise for supporting brain health, significant limitations constrain their clinical translation. The microbiome response to metformin varies widely across individuals and is influenced by diet, age, metabolic status, and comorbidities, making therapeutic effects difficult to predict. Metformin’s pleiotropic mechanisms also complicate interpretation. Improvements in cognition or neuroinflammation may reflect direct metabolic or vascular effects rather than microbiome modulation. Long-term use can cause gastrointestinal side effects and vitamin B12 deficiency, which may adversely affect neurological function. Moreover, evidence linking metformin-induced microbiome changes to cognitive outcomes is primarily based on preclinical or observational data, with few controlled clinical trials.

The gut microbiome is highly dynamic and shaped by numerous external factors, making it challenging to achieve consistent, durable therapeutic shifts. Current interventions demonstrate variable efficacy, and the specific microbial signatures most relevant to cognitive health remain unclear. Many studies focus on microbial composition rather than function, limiting mechanistic insight. Additionally, substantial interindividual variability and the complex, bidirectional gut–brain axis reduce generalizability and hinder translation to routine clinical practice.

## 13. Future Directions

Metagenomics, metabolomics, and transcriptomics should be employed in future studies to delineate specific microbial signatures linked to metabolic and neurocognitive outcomes and to evaluate the effects of microbiota-targeted therapies on enhancing gut–brain axis communication and ameliorating metabolic dysfunction. Furthermore, further investigation into mitochondrial signaling mechanisms, bile acid receptor interactions, and SCFA-mediated pathways is needed to identify novel biomarkers for the diagnosis and treatment of metabolic and neurodegenerative disorders.

## Figures and Tables

**Table 1:** Role of gut microbiota.

Microbiota	Role	Mechanism	Key References
*Akkermansia municipia*	Enhances mucosal barrier, improves insulin sensitivity	Mucin degradation, increase SCFA, and tight-junction reinforcement	de la Cuesta-Zuluaga et al., 2017; Sun et al., 2018 [13,14]
*Bacteroidetes*	Maintain immune-metabolic balance	Ferment carbohydrates, producing propionate, acetate	Thursby & Juge, 2017; de Vos et al., 2022 [3,17]
*Firmicutes*	Produce butyrate, regulate the barrier, and reduce inflammation	Butyrate activates FFAR2/3; supports epithelial junctions	Silva et al., 2020; Kim, 2023 [18,19]
*Bifidobacterium* spp.	Produce folate, GABA, and strengthen gut barrier	Folate biosynthesis, GABA synthesis, and increased anti-inflammatory signaling	Pompei et al., 2007; LeBlanc et al., 2013 [20,21]
*Lactobacillus* spp.	Generate GABA, acetylcholine, and protect mucosa	Neurotransmitter synthesis, immune modulation	Lyte, 2013; Holzer & Farzi, 2014 [22,23]
*Escherichia* spp.	Influence CNS via serotonin, noradrenaline	LPS and monoamine production	Lyte, 2013; Morais et al., 2021 [12,23]
*Clostridial* spp.	Anti-inflammatory butyrate producers	Butyrate activates AMPK, FFAR2/3, and lowers inflammation	den Besten et al., 2013; Mishra et al., 2020 [24,25]
*Bacteroides* spp.	Modulate bile acid metabolism, glucose homeostasis	Convert bile salts to DCA/LCA; activate FXR/TGR5	Ridlon et al., 2016; Lun et al., 2024 [26,27]
*Faecalibacterium prausnitzii*	Anti-inflammatory, butyrate-producing commensal	Increases IL-10; inhibits NF-κB	Silva et al., 2020 [18]
*Streptococcus thermophilus*	Preserves epithelial integrity	Prevents TNF-α–induced barrier loss	Resta-Lenert & Barrett, 2006 [28]
*Lactobacillus acidophilus*	Maintains junction integrity and reduces permeability	Prevents IFγ-induced cytokine disruption	Virk et al., 2024 [29]

AMPK: AMP-activated protein kinase—ANS: Autonomic nervous system—BBB: Blood–brain barrier—CNS: Central nervous system—ECCs: Enterochromaffin cells—EECs: Enteroendocrine cells—ENS: Enteric nervous system—FFAR2/3: Free fatty acid receptors 2 and 3—FXR: Farnesoid X receptor—GABA: Gamma-aminobutyric acid—GI: Gastrointestinal—GLP-1: Glucagon-like peptide 1—HPA: Hypothalamic–pituitary–adrenal axis—IFγ: Interferon gamma—IL: Interleukin 10—LPS: Lipopolysaccharide—NF-κB: Nuclear factor kappa-B—PYY: Peptide YY—SCFAs: Short-chain fatty acids—TGR5: Takeda G protein-coupled receptor 5—TLR-4: Toll-like receptor 4.

**Table 2:** Effects of Metformin on Microbiome Regulation.

Design	Sample/Model	Intervention/Exposure	Outcomes/Findings	Reference
Case–Control	4-wk-old C57BL/6 mice on HFD or NCD for 8 wks	Metformin (300 mg/kg/day) PO for 6 weeks	Increase *Akkermansia* spp., and ileal goblet cells; improved glucose tolerance. Oral *A. muciniphila* mimicked metformin benefits	Shin NR et al., 2014 [131]
Case–Control	6-wk-old male C57BL/6N mice on HFD or NCD for 23 wks	(1) Metformin (250 mg/Kg/day) for 16 wks. (2) FMT pooled from all mice (20 mg) PO for 4 wks. (3) A. *muciniphila* EVs (20 µg/day) PO for 5 wks.	Metformin increases *Akkermansia*, and *Bacteroides*. It reduced epididymal fat IL-1β/IL-6. FMT and *A. muciniphila* EVs improved weight, glycemia, and lipids.	Lee H et al., 2018 [132]
Multi-host Metagenomic study	T2D metformin-naïve people (n = 22). Microbiota-depleted mice on HFD. *Fxr* knockout control mice on HFD	Humans: Metformin (1000 mg b.i.d) PO for 3 days. Mice: Live or dead 108 CFU *B. fragilis* PO twice weekly with or without metformin (200 mg/kg/d) after 3 d of antibiotics cocktail	*B. fragilis*–GUDCA–intestinal FXR axis as mediator of metformin’s metabolic benefits	Sun L et al., 2018 [14]
Nematode model of host–microbe–drug interaction	*C. elegans* co-cultured with *E. coli*	Metformin	Lifespan extension via altered microbial folate/methionine metabolism	Cabreiro F et al., 2013 [133]
Cross-sectional metagenomics	784 human gut metagenomes (T2D and controls)	Metformin use (exposure)	Metformin strongly shapes T2D microbiome signatures (increases Escherichia, SCFA-related functions), confounding prior disease-only signals	Forslund K et al., 2015 [16]
Cross-sectional cohort	PWD (n = 14 on Metformin) and people without T2D (n = 84)	Metformin use (exposure)	Metformin is associated with increased *A. muciniphila* and multiple SCFA-producing microbiota	de la Cuesta et al., 2017 [13]
Double-blind RCT—Multi-host study	Treatment-naïve T2D adults (n = 40). Male Swiss germ-free Webster	Metformin (Initiation: 425 mg/d—Maintenance: 1700 mg/d) or placebo for 4 mo—A placebo subgroup switched to metformin. FMT (200 μL M0/M4 fecal slurry) PO to germ-free mice on HFD for 18 days.	Metformin produced large microbiome shifts; FMT from metformin-treated donors improved glucose tolerance in mice	Wu H et al., 2017 [15]
3-parallel-arm Randomized trial	Overweight/obese cancer survivors (n = 121)	Metformin (up to 2000 mg PO) vs. behavioral weight loss vs. self-directed care for 12 mo	Metformin (not weight loss) altered microbiome composition and SCFA levels; both increased acetate, linked to lower fasting insulin	Mueller NT et al., 2021 [134]
Non-blinded, one-arm crossover	Healthy young men (n = 27)	Metformin up-titrated to 1 g b.i.d	Reversible changes in 11 genera (Increase Escherichia/Shigella, Bilophila; decrease Intestinibacter, Clostridium); baseline microbiota predicted GI side effects	Bryrup T et al., 2019 [135]

b.i.d: twice daily—EVs: Extracellular vesicles—FMT: Fecal microbiota transplantation—FXR: Farnesoid X receptor—GLP-1: Glucagon-Like Peptide 1—GUDCA: bile acid glycoursodeoxycholic acid—HFD: High-Fat Diet—IL: Interleukin—mo: month—n: number—NCD: Normal-Chow Diet—PO Per Os—PWD: People with Diabetes—SCFA: Short-Chain Fatty Acid—spp.: Species—T2D: Type 2 diabetes—Wk: Week.

## Data Availability

No new data were created or analyzed in this study.

## Abbreviations

The following abbreviations are used in this manuscript:

AgRP	Agouti-related peptide
AMPs	Antimicrobial proteins
AMPK	AMP-activated protein kinase
ANS	Autonomic nervous system
BBB	Blood–brain barrier
b.i.d	Twice daily
CNS	Central nervous system
ECCs	Enterochromaffin cells
EECs	Enteroendocrine cells
ENS	Enteric nervous system
EVs	Extracellular vesicles
FFA2	Free fatty acid 2
FFAR2/3	Free fatty acid receptors 2 and 3
FGF19	Fibroblast growth factor 19
FMT	Fecal microbiota transplantation
FXR	Farnesoid X receptor
GABA	Gamma-aminobutyric acid
GALT	Gut-associated lymphoid tissues
GBA	Gut–brain axis
GI	Gastrointestinal
GLP-1	Glucagon-like peptide-1
GUDCA	Bile acid glycoursodeoxycholic acid
HFD	High Fat Diet
hNSCs	Human neural stem cells
HPA	Hypothalamic–pituitary–adrenal axis
IECs	Intestinal epithelial cells
IFγ	Interferon gamma
IL	Interleukin
LPS	Lipopolysaccharide
NCD	Normal chow diet
NPY	Neuropeptide Y
NF-κB	Nuclear factor kappa-B
PG	Peptidoglycan
PO	Per Os
POMC/α-MSH	proopiomelanocortin/alpha-melanocyte-stimulating hormone
PWD	People with Diabetes
PYY	Peptide YY
SCFAs	Short-chain fatty acids
sIgA	Secretory immunoglobulin A
Spp	Species
T2D	Type 2 diabetes
TGF	Transforming growth factor
TGR5	Takeda G protein-coupled receptor 5
TLRs	Toll-like receptors
